# Combined use of mitochondrial and nuclear genetic markers further reveal immature marine turtle hybrids along the South Western Atlantic

**DOI:** 10.1590/1678-4685-GMB-2019-0098

**Published:** 2020-04-27

**Authors:** Cíntia Brito, Sibelle Torres Vilaça, Ana Luzia Lacerda, Rodrigo Maggioni, Maria Ângela Marcovaldi, Gabriela Vélez-Rubio, Maíra Carneiro Proietti

**Affiliations:** 1 Universidade Federal do Rio Grande (FURG), Instituto de Oceanografia (IO), Laboratório de Ecologia Molecular Marinha, Rio Grande, RS, Brazil.; 2 Trent University, Environmental and Life Sciences Graduate Program, Peterborough, Canada.; 3 Universidade Federal do Ceará (UFC), Instituto de Ciências do Mar (LABOMAR), Fortaleza, CE, Brazil.; 4 Instituto Chico Mendes de Conservação da Biodiversidade (ICMBio), Projeto TAMAR, Salvador, BA, Brazil.; 5 Fundação Pró-TAMAR, Salvador, BA, Brazil.; 6 Asociación Civil Karumbé, Montevideo, Uruguay.; 7 Universidade de la República, Centro Universitario Regional del Este (CURE), Rocha, Uruguay.; 8 Department of Evolutionary Genetics, Leibniz Institute for Zoo and Wildlife Research, Berlin, Germany.

**Keywords:** Hybridization, Cheloniidae, Genetic markers, Hybrid distribution, Conservation

## Abstract

Marine turtle hybridization is usually sporadic and involves reports of only a few individuals; however, Brazilian populations have high hybridization rates. Here we investigated the presence of hybrids in morphologically identified immature hawksbills (*Eretmochelys imbricata*) along the South Western Atlantic (SWA). We sequenced one mitochondrial (D-Loop) and three nuclear DNA (RAG1, RAG2, and CMOS) markers to better understand the patterns and characteristics of hybrids. We identified 22 hybrids (n = 270), 11 of them at the extreme South of the SWA. Uruguay had the highest hybrid frequency in the SWA (~37.5%) followed by southern Brazil with 30%. These are common areas for loggerheads (*Caretta caretta*) but uncommon for hawksbills, and these hybrids may be adopting the behavior of loggerheads. By analyzing nuclear markers, we can infer that 50% of the sampled hybrids are first generation (F1) and 36% are the result of backcrosses between hybrids and pure *E. imbricata* (> F1). We also report for the first time immature *E. imbricata* x *Lepidochelys olivacea* hybrids at the Brazilian coast. Considering the high frequency of hybrids in the SWA, continuous monitoring should be performed to assess the fitness, genetic integrity, and extent of changes in the gene pools of involved populations.

## Introduction

Hybridization can be defined as the production of offspring by the crossbreeding between genetically different populations or species (Harrison, 1990). At least 25% of plant species and 10% of animal species are involved in hybridization processes and potential introgression (Mallet, 2005). According to Rieseberg and Wendell (1993), introgression can be defined as “the incorporation of genes from one set of different populations into another, i.e. the incorporation of external alleles into a new, reproductively integrated population system”. These processes are considered natural in evolution, and continuous events of interspecific and intergeneric hybridization may lead to the appearance of new species (e.g. 50-70% of angiosperms; Whitham *et al.*, 1991). However, hybridization can also be a consequence of anthropogenic factors such as decreasing population sizes, introduction of non-native species and modification of habitats, which may result in the extinction of local species, subspecies and populations (Allendorf *et al.*, 2001).

Hybridization is a common phenomenon in different vertebrate groups, such as birds (Crochet *et al.*, 2003), fish (Rosenfield *et al.*, 2004), and marine mammals - the order Cetacea, for example, has records of hybrids in 20% of species that make up the group (Crossman *et al.*, 2016). In marine turtles, the occurrence of hybrids has already been reported between several species of the Cheloniidae family, especially the closely related species olive ridley (*Lepidochelys olivacea*), loggerhead (*Caretta caretta*), and hawksbill (*Eretmochelys imbricata*) (Bowen and Karl, 2007; Naro-Maciel *et al.*, 2008). It is possible that this interbreeding occurs due to the lack of reproductive barriers (Seminoff *et al.*, 2003) and a chromosomal compatibility between the different species (Karl *et al.*, 1995).

Most data on hybridization in nature comes from morphological evaluation of organisms (Mallet, 2005); however, this alone is insufficient to adequately identify and characterize hybrids, since some hybrids do not present mixed morphology. In marine turtles, the first signs of hybridization were observed in animals with intermediate diagnostic characteristics between two species (Wood *et al.*, 1983), and the first molecular observation of hybrids was done in Brazil by Conceição *et al.* (1990), using isozymes. With the advance of molecular tools, genetic analyses have been increasingly used to detect and understand this hybridization process (e.g. Vilaça *et al.*, 2012). Mitochondrial (mtDNA) and nuclear (nDNA) markers may therefore aid in the identification of hybrids even when individuals do not have evidence of hybridization observable through morphology.

In most species, mtDNA is maternally inherited. As a result, relying only on mitochondrial information may be misleading when validating morphological observations; for example, if a first-generation (F1) or any subsequent generation (>F1) hybrid shows similar morphological characteristics to the species determined through mtDNA, hybridization cannot be detected. In contrast, nDNA is inherited from the two progenitors, allowing the identification of genes of different species even when the hybrid has the morphological characteristics and mtDNA of only one (Vilaça and Santos, 2013). Thus, the combined use of mtDNA and nDNA markers assists in hybrid identification, allowing a better evaluation of their distribution and frequency. In addition, it is possible to evaluate the number of generations over which hybridization has been occurring, as well as the occurrence of introgression (i.e., whether the individual is the result of a cross between pure parent species (F1 generation), hybrids (F2) or a backcross between a hybrid and one of the pure parent species) (Sunnucks, 2000). Identifying the degree of introgression between species is important in assessing possible losses of locally adapted genes and population fitness (Allendorf *et al.*, 2001). In addition, according to Payseur and Rieseberg (2016), understanding the connection between geographic distribution/gene flow of hybrids and the emergence of new species is fundamental. Considering the hybridization events and species involved, the decision of which individuals and populations should be protected is complex (Wayne and Shaffer 2016), and caution is needed when establishing conservation strategies.

Hybridization events in marine turtles are usually sporadic and involve reports of one or a few individuals (see Table 1); however, Brazilian populations have high rates, which may be due to the endangered status of these animals (IUCN, 2018). At the Bahia state rookery, Lara-Ruiz *et al.* (2006) observed through mtDNA that, among *E. imbricata* females analyzed (n = 119), 42% were actually hybrids with *C. caretta* and 2% hybrids with *L. olivacea*. When analysing 204 samples of *C. caretta* nesting females at four rookeries (Rio de Janeiro, Espírito Santo, Bahia and Sergipe states), Reis *et al.* (2010) observed that 14 out of 51 females from Sergipe were hybrids with *L. olivacea*, with no record for the other rookeries. Occurrences of *C. caretta* and *E. imbricata* immature hybrids have also been reported in Uruguay and Argentina (Alvarez-Varas *et al.*, 2016; Prosdocimi *et al.*, 2014). The occurrence of *E. imbricata* along the coast of Uruguay is low, with only three individuals registered during twelve years of monitoring by the NGO Karumbé (Vélez-Rubio *et al.*, 2013). However, during these surveys several turtles with inconclusive morphology (i.e. possible hybrids) have been observed (A. Fallabrino, personal communication).

**Table 1:** Hybridization events reported between Cheloniidae marine turtles, the respective species, number of observed hybrids and country.

Species A	Species B	No. identified hybrids	Country	References
*Caretta caretta*	*Lepidochelys kempii*	1	USA	Karl et al., 1995
*Caretta caretta*	*Chelonia mydas*	4	Brazil	Karl et al., 1995
		1	Canada	James et al., 2004
		3	USA	Komoroske et al., 2019
*Caretta caretta*	*Lepidochelys olivacea*	14	Brazil	Reis et al., 2010
*Eretmochelys imbricata*	*Caretta caretta*	2	USA	Karl et al., 1995
		50	Brazil	Lara-Ruiz et al., 2006
		1	Brazil	Conceição et al., 1990
		4	Brazil	Proietti et al., 2012
		34	Brazil	Soares et al., 2017
		10	Brazil	Bass et al., 1996
		1	USA	Komoroske et al., 2019
		2	Argentina	Prosdocimi et al., 2014
*Eretmochelys imbricata*	*Chelonia mydas*	1	Suriname	Karl et al., 1995
		1	Mexico	Seminoff et al., 2003
		1	Peru	Kelez et al., 2016
*Eretmochelys imbricata*	*Lepidochelys olivacea*	2	Brazil	Lara-Ruiz et al., 2006

The Brazilian coast has the highest known rate of hybridization in the world between four sea turtle species. The hybridization pattern involving the most common species in Brazil - *E. imbricata*, *C. caretta* and *L. olivacea* - was investigated by Vilaça *et al.* (2012) in samples obtained along the coast using 12 nuclear markers. These authors identified *L. olivacea* hybrids with *C. caretta* and *E. imbricata* as being first generation (F1). In contrast, some *C. caretta* and *E. imbricata* hybrids showed evidence of backcrosses with pure parent species, indicating a longer process or higher survival of offspring. They also suggested that hybridization events at the region have been occurring for at least 40 years (i.e., around two generations), and may be a result of the historical population decline experienced by both species due to exploitation of eggs and female turtles (Santos *et al.*, 2011).

In Brazil, *E. imbricata* reproductive areas overlap spatially and temporally with those of *C. caretta*, with both species reproducing at the Northeast coast and presenting highest nesting concentrations at Bahia and Sergipe states (Marcovaldi *et al.*, 2007; Marcovaldi and Chaloupka, 2007). The *C. caretta* reproductive season begins in September and ends in February (Santana *et al.*, 2011), and *E. imbricata* breeding begins in November and extends until March (Marcovaldi *et al.*, 2011). This overlap, together with the population depletion suffered by the species, may contribute to the occurrence of hybridization events. Proietti *et al.* (2014a) and Vilaça *et al.* (2012) showed that the hybridization happening in Brazil has a gender bias, and that the encounter of male *E. imbricata* with female *C. caretta* would be favored by the larger population size of loggerhead turtles, in conjunction with a temporal overlap at the peak of reproductive season. *E. imbricata* begins to reproduce near the *C. caretta* reproductive peak (November-December), leading to the encounter of male *E. imbricata* with females of both species. In contrast, since the reproductive peak of *E. imbricata* is after *C. caretta*, most *C. caretta* have probably left the area when it presents higher numbers of *E. imbricata* females, reducing the probability of their reproduction (Soares *et al.*, 2018).

Immature animals resulting from the hybridization process in Brazil were reported for the first time by Proietti *et al.* (2014a, b), who analyzed 157 *E. imbricata* along the Brazilian coast and identified four individuals at Cassino beach (Rio Grande do Sul state) with a *C. caretta* haplotype (CCA4.2). This is an unusual area for *E. imbricata*, which occupies preferentially tropical regions of the oceans, associated with coral reefs (León and Bjorndal, 2002; Mortimer and Donnelly, 2008); on the other hand, *C. caretta* is common at temperate latitudes, occurring frequently at the region (Monteiro *et al.*, 2016). The high frequency of immature hybrids found at Cassino shows that the distribution of these animals may present a spatial pattern, with preference for areas used more by *C. caretta*. Adult hybrids also show differential habitat use: Marcovaldi *et al.* (2012) tracked pure and hybrid *E. imbricata x C. caretta* nesting females from Bahia, and observed that they used distinct foraging areas. While pure females migrated to their respective species areas along the Brazilian coast, hybrids migrated predominantly to the northeast Brazilian coast, a *C. caretta* feeding area, although some hybrids also migrated south to an *E. imbricata* feeding area (Lima *et al.*, 2013).

The effect of hybridization on marine turtle fitness and reproduction output was investigated for the first time by Soares *et al.* (2017), who compared factors associated with reproductive success of hybrid and pure *E. imbricata* and *C. caretta* females nesting at Bahia. Only 1% of females identified morphologically as *C. caretta* were hybrids, while more than half of the females identified as *E. imbricata* presented a *C. caretta* haplotype. This reaffirms the prevalence of crosses between *E. imbricata* males and *C. caretta* females. Based on the analysed reproductive parameters (number of eggs, emergence success, incubation period, number of hatchlings per nest, number of nests per year), hybrid females apparently do not have different reproductive outputs when compared to pure parent species.

Considering the high occurrence of hybrids in the South Western Atlantic (SWA), the potential impacts of this phenomenon on marine turtle populations, and the paucity of studies on the characteristics of hybrid offspring originating from Brazilian populations, the goal of this study was to investigate hybridization in immature turtles along the SWAChange to: SWA, using molecular methods. Through the combined analysis of mtDNA (control region - D-Loop) and nDNA (RAG1, RAG2 and CMOS) markers, we evaluated if: 1) the use of multiple markers enhances the detection of immature hybrids; 2) immature hybrid turtles present preference for certain foraging areas; and (3) the identified immature hybrid turtles are first generation (F1), the result of crossing between hybrids (F2), or of introgression between hybrids and pure parent species (>F1).

## Materials and Methods

### Sampling

**Figure 1:**
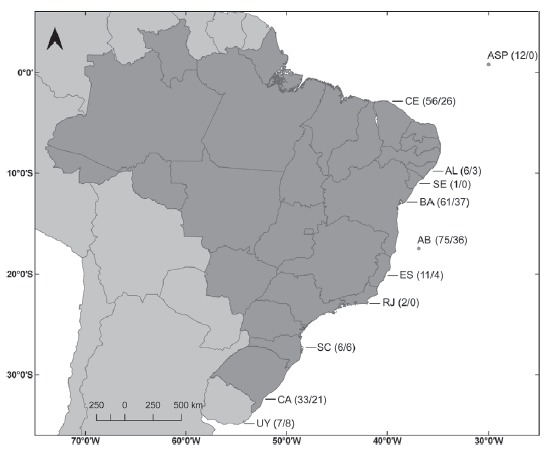
Sampling sites in the South Western Atlantic, indicating the number of individuals analyzed for mtDNA/nDNA. ASP São Pedro and São Paulo Archipelago; AB Abrolhos Archipelago; AL Alagoas; CA Cassino; CE Ceará, BA Bahia; ES Espírito Santo; RJ Rio de Janeiro; SC Santa Catarina; SE Sergipe; UY Uruguay.

A total of 270 skin and/or muscle samples were obtained from the anterior flippers or inguinal area of immature hawksbills (Curved Carapace Length - CCL - from 13 to 111 cm) incidentally captured by fisheries, stranded on the beach, or intentionally caught in dives along the coast of Brazil and Uruguay. In Brazil, sampling was done at the São Pedro and São Paulo (ASP) and Abrolhos (AB) Archipelagos, along the coast of Alagoas (AL), Bahia (BA), Ceará (CE), Espírito Santo (ES), Santa Catarina (SC) and Sergipe (SE) states, and Cassino beach (Rio Grande do Sul state (Figure 1). Projeto Tamar - Fundação Tamar and Centro Tamar-ICMBio, provided samples from BA, CE, ES and SE; Instituto Biota de Conservação provided samples from AL; and Núcleo de Educação e Monitoramento Ambiental (NEMA) provided samples from Cassino. The NGO Karumbé provided samples from the coast of Uruguay (UY). All samples had their mtDNA characterized (part of which were described in Proietti *et al.*, 2014b); for nDNA 141 samples were analyzed, prioritizing sites where hybrids had been identified through mtDNA.

### Molecular analyses

Genomic DNA was extracted using a commercial extraction kit (Qiagen DNEasy Extraction Kit), according to the manufacturer’s protocol. Fragments of the mtDNA control region (~850 bp) were amplified via Polymerase Chain Reaction (PCR) using primers LCM15382 and H950 (Abreu-Grobois *et al.*, 2006), under the following conditions: 5’ at 94 °C; 36 cycles of 30 s at 94 °C, 30 s at 50 °C 1min at 72 °C; and a final extension of 10 min at 72 °C. Fragments of nDNA were amplified using primers previously described by Vilaça *et al.* (2012), for three different genes: oocyte maturation factor (CMOS - 601 bp and 13 polymorphic sites), and two somatic recombination activating genes (RAG1 - 368 bp and 10 polymorphic sites; RAG2 - 620 bp and 8 polymorphic sites). These markers were shown to be species-specific and effectively differentiate various sea turtle species and their hybrids (Vilaça *et al.*, 2012). PCR cycle conditions were: 5’ denaturation at 94 °C; 35 cycles consisting of 30 s at 94 °C; 1 min under specific annealing temperatures (62.5 °C for RAG1 and 67 °C for RAG2 and CMOS), 1 min at 72 °C; and a final extension of 10 min at 72 °C. Each nDNA marker was amplified for a distinct number of samples, since it was not possible to amplify the three markers for all analysed individuals.

Amplified products were purified with purification kits (GE Healthcare Illustra GFX Purification kit) and quantified by spectrophotometry using a BioDrop μLITE. The purified products were then sequenced at Macrogen (http://dna.macrogen.com/eng/). Quality analysis of the sequences obtained for both mtDNA and nDNA was performed with Chromas 2.6.5 software (https://technelysium.com.au). The mtDNA fragments were aligned using the Clustal W tool (Larkin *et al.*, 2007) implemented in BioEdit 7.0.9 (Hall, 1999). Sequences were cropped to 740 bp and classified according to GenBank (https://www.ncbi.nlm.nih.gov/) and the Archie Carr Center for Sea Turtle Research database (http://accstr.ufl.edu/resources/mtdna-sequences/). Since all individuals sequenced were morphologically identified as *E. imbricata*, those that had mtDNA of other species were considered as hybrids based on this marker. For nDNA, sequence analysis was performed using the PHASE tool of DNAsp software (Librado and Rozas, 2009) to identify the haplotype of each allele of the analysed markers. The nDNA haplotypes previously described by Vilaça *et al.* (2012; Table S1) were used as prior information.

To characterize hybridization and introgression, we followed the considerations presented by Vilaça *et al.* (2012): F1 hybrids exhibit for all loci alleles derived from different species, e.g., a *C. caretta* x *E. imbricata* F1 hybrid shows for all loci one *C. caretta* and one *E. imbricata* allele; introgressed animals (>F1) show for one or more loci two alleles of the same species, e.g. for RAG1 the hybrid individual presents two alleles exclusive to *C. caretta*.

### Data analyses

To update the frequency of occurrence and distribution of immature hybrids based on mtDNA, we grouped the sequences of the 112 samples analyzed in this work with the 158 presented by Proietti *et al.* (2014b). Based on the haplotype identified for each sample, a haplotype network was built using PopArt (Leigh and Bryant, 2015), with the Median-Joining method (Bandelt *et al.*, 1999).

Bayesian clustering methods were used to detect the level of introgression through STRUCTURE (Pritchard *et al.*, 2000) and NewHybrids (Anderson and Thompson, 2002), based on the nDNA markers. These programs require information on the characteristic haplotypes of each species (coded as bi-allelic genotypes) to infer the ancestry/admixture of the individuals analyzed, and we therefore used as input the database generated by Vilaça *et al.* (2012). STRUCTURE analysis was performed assuming non-correlated allele frequencies in the admixture model, with a burn-in of 100,000 and 1,000,000 randomizations collected via Markov Chain Monte Carlo (MCMC), with a K value ranging from 1 to 10, with 5 independent iterations. The best K was chosen using the online tool CLUMPAK (Kopelman *et al.*, 2015), according to the Evanno method (Evanno *et al.*, 2005).

The NewHybrids analysis was implemented considering six classes for identification: two classes for pure species (Ei and Cc), and four for hybrids - first generation (F1), second generation (F2), and backcrosses with pure species (F1xEi and F1xCc) - since no hybrid above F2 could be statistically detected by this method (Anderson and Thompson, 2002). This analysis was performed with a burn-in of 10,000 and 1,000,000 randomizations collected via the Markov Chain (MCMC). NewHybrids uses a model that considers only two pure parent species, so in this case we used samples identified by Vilaça *et al.* (2012) as being of *E. imbricata* and *C. caretta*, excluding from the analysis the other species of the database. Thus, samples “CE32" and ”CE43" were not considered in this analysis, since they possessed mtDNA of *L. olivacea*.

## Results

### mtDNA

**Figure 2:**
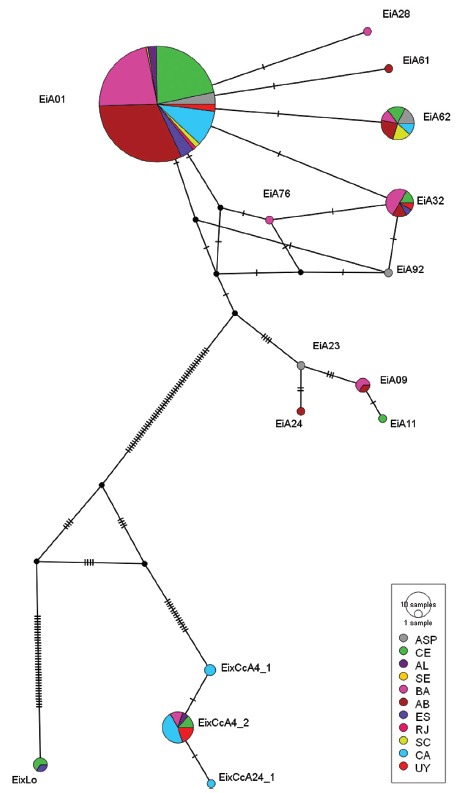
mtDNA (D-Loop) haplotype network constructed based on the Median-Joining method. Circles represent each of the identified haplotypes, size corresponds to the frequency of occurrence of the haplotype, and color represents the sampling location. Dashes between haplotypes represent the number of distinct bases between them.

**Figure 3:**
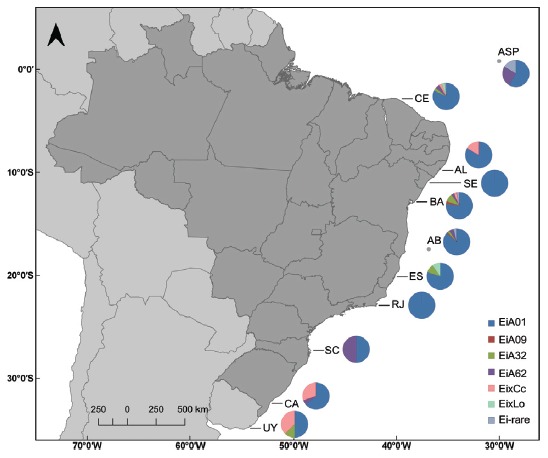
Geographic distribution of D-Loop haplotypes found in the mtDNA analysis along the South Western Atlantic. ASP São Pedro and São Paulo Archipelago; (n = 12); AB Abrolhos Archipelago (n = 75); AL - Alagoas (n = 6); CA - Cassino (n = 33); CE - Ceará (n = 56), BA - Bahia (n = 61); ES - Espírito Santo (n = 11); RJ Rio de Janeiro (n = 2); SC Santa Catarina (n = 6); SE - Sergipe (n = 1); UY - Uruguay (n = 7).

We analysed mtDNA from a total of 270 individuals distributed along the 11 collection sites, based on the 740bp fragments of the control (D-Loop) region. Fifteen haplotypes were identified (Figure 2), with eleven being characteristic of *E. imbricata* and four of other species. The *E. imbricata* haplotype distribution (Figure 3) showed a predominance of haplotype EiA01, found in all areas and with a total frequency of 78%. The less frequent haplotypes were EiA62 (6%), EiA32 (4%), EiA09 (1%), and rare haplotypes, with only one occurrence each, were EiA11, EiA23, EiA24, EiA28, EiA61, EiA76 and EiA92.

Of the haplotypes of other species, three are specific to *C. caretta* (CC-A4.2 - 5.6%, CC-A4.1 - 0.7% and CC-A24.1 - 0.4%), and one to *L. olivacea* (Haplotype F - 1.1%), all of which were previously shown to occur in Brazil’s nesting grounds (Bowen *et al.*, 1998; Shamblin *et al.*, 2014). Two hybrid turtles with *L. olivacea* haplotypes were found in Espírito Santo and one in Ceará, and all three hybrids had haplotype F. Of the 17 hybrids with *C. caretta*, observed at five sites, 10 were found in Cassino, one in Alagoas, two in Bahia, two in Ceará, and two in Uruguay. These hybrids showed a predominance of the CC-A4.2 haplotype, with one occurring in Alagoas (out of a total of 6), two in Bahia (total n = 61) and two in Ceará (total n = 56). Interestingly, Cassino showed a high frequency of hybrids with three distinct *C. caretta* haplotypes - CC-A4.2 (21%), CC-A4.1 (6%) and CC-A24.1 (3%) - and the presence of the most frequent *E. imbricata* haplotypes - EiA01 (64%) and EiA62 (6%) (total n = 33). Uruguay presented two *E. imbricata* haplotypes - EiA01 (50%) and EiA32 (12.5%) - and displayed only one *C. caretta* haplotype, CCA4.2 (37.5%) (total n = 8).

### nDNA

Six haplotypes were found for RAG1 (n = 126): Hap3, species-specific of *E. imbricata*; Hap1 and Hap4, shared between *E. imbricata* and *L. olivacea*; Hap2, species-specific of *C. caretta*; Hap8, species-specific of *C. mydas*; and a previously unidentified haplotype (named Hap10, Table S1). For RAG2 (n = 89), four haplotypes were found: Hap5, species-specific of *E. imbricata*; Hap2, species-specific of *C. caretta*; Hap6, species-specific of *C. mydas*; and a previously unidentified haplotype (named Hap7, Table S1). For CMOS (n = 35), six haplotypes were found: Hap3, Hap5, Hap9 and Hap10, species-specific of *E. imbricata*; and Hap1 and Hap2, species-specific of *C. caretta*.

In general, most samples were homozygotes (RAG1 = 83%, RAG2 = 94%, CMOS = 63%), while heterozygotes were in most cases hybrid individuals that had alleles of different species. The exception was RAG1, in which 11 of the 21 heterozygotes had a haplotype that can be found in both *E. imbricata* and *L. olivacea*, and therefore it was not possible to determine if they were hybrids or pure *E. imbricata*. The CMOS marker was the only one to identify eleven heterozygotes among the pure individuals, i.e., individuals that presented two distinct but species-specific *E. imbricata* haplotypes in each of the alleles.

**Figure 4:**
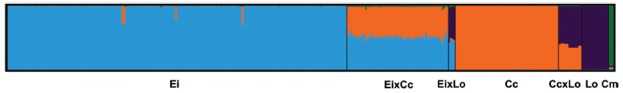
Cluster analysis performed in STRUCTURE for three nDNA markers (RAG1, RAG2 and CMOS). X-axis represents each of the individuals analysed, y-axis is the estimated mixture ratio of each of the parent species in the composition of these individuals. Species abbreviation and colors: blue, *E. imbricata* (Ei); orange, *C. caretta* (Cc); purple, *L. olivacea* (Lo); green, *C. mydas* (Cm).

In STRUCTURE, the best K (K = 4) separated *E. imbricata* (Ei), *C. caretta* (Cc), *L. olivacea* (Lo) and *C. mydas* (Cm) in different groups (Figure 4), and hybrids that had alleles of two or more distinct species were also identified. Of the 17 *E. imbricata* x *C. caretta* hybrids identified through mtDNA, for five samples it was not possible to amplify any of the nuclear markers used in this work. Therefore, these five samples were not included in the subsequent analysis. Clustering results showed that *E. imbricata* x *C. caretta* (Ei x Cc) hybrids presented different degrees of hybridization. Surprisingly, two individuals (CA23 from Cassino and PF06 from Bahia), classified as *E. imbricata* through morphological analysis and mtDNA, presented alleles of *C. caretta* and therefore appear in the ‘Ei’ group with ‘Cc’ components. These hybrids have the particularity of having mtDNA and morphology of *E. imbricata*, a condition observed at low frequency (1%) by Vilaça *et al.* (2012). Therefore, these two individuals might be: 1) the result of the cross between a female *E. imbricata* and a male *C. caretta* (rare occurrence), and are therefore F1; or 2) a cross between a female *E. imbricata* female and a male Ei x Cc hybrid, hence generation >F1. Based only on the available data, it was not possible to distinguish between the two possibilities.

For the three individuals identified as hybrids with *L. olivacea* (Ei x Lo) through mtDNA, it was possible to amplify only one nDNA marker (RAG1) in two individuals, and both presented Hap1, shared between *E. imbricata* and *L. olivacea*. Therefore, inference on the degree of hybridization of these individuals was not possible since these samples were not included in the STRUCTURE analysis, and they were categorized as hybrids based only on mtDNA. In addition to the *E. imbric*ata x *C. caretta* hybrids previously identified by mtDNA, it was possible to find two additional hybrids (CA23 and PF06) that presented mtDNA of *E. imbricata* and nDNA with one allele of each of the pure parent species, for all amplified markers.

**Figure 5:**
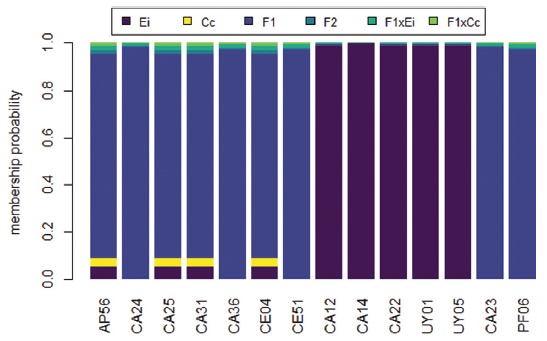
Assignment probability model for the 12 E. imbricata x C. caretta hybrids identified by mtDNA analysis (AP56 UY05) and two additional hybrids identified only through nDNA (CA23 and PF06). The y-axis represents the probability that each individual belongs to each of the six categories presented (Ei, Cc, F1, F2, F1xEi e F1xCc).

NewHybrids correctly identified all pure individuals as *E. imbricata* (*p* > 0.98, Figure 5). Seven hybrids (AP56, CA24, CA25, CA31, CA36, CE04, CE51) were classified as F1 (*p* > 0.90), and had in all loci a species-specific haplotype of the parent species with no evidence of introgression. Five other hybrids (CA12, CA14, CA22, UY01, and UY05) were not classified as hybrids since they had species-specific *E. imbricata* haplotypes in all loci, despite having *C. caretta* mtDNA, thus demonstrating signs of introgression with *E. imbricata.* An information summary for all hybrids identified at the South Western Atlantic, with mtDNA haplotype, parental species involved, and inference on generations, can be seen in Table 2.

**Table 2:** Immature sea turtle hybridsImmature sea turtle hybrids identified at the South Western Atlantic, with sampling area, mtDNA haplotype, species involved, and inference on hybrid classes. AL - Alagoas, BA - Bahia, CA - Cassino, CE - Ceará, ES - Espirito Santo, UY - Uruguay.

Sample	Area	Haplotype	mtDNA	nDNA	F - M*	Classes
AL02	AL	CCA4.2	EixCc	-	Cc - Ei	-
AP56	BA	CCA4.2	EixCc	EixCc	Cc - Ei	F1
PF06	BA	EiA01	Ei	EixCc	Ei - Cc or Ei - EixCc	F1 or F1xEi
SA10	BA	CCA4.2	EixCc	-	Cc - Ei	-
CA04	CA	CCA4.2	EixCc	-	Cc - Ei	-
CA12	CA	CCA4.2	EixCc	Ei	EixCc - Ei	F1xEi
CA14	CA	CCA4.2	EixCc	Ei	EixCc - Ei	F1xEi
CA22	CA	CCA4.2	EixCc	Ei	EixCc - Ei	F1xEi
CA23	CA	EiA01	Ei	EixCc	Ei - Cc or Ei - EixCc	F1 or F1xEi
CA24	CA	CCA4.2	EixCc	EixCc	Cc - Ei	F1
CA25	CA	CCA4.2	EixCc	EixCc	Cc - Ei	F1
CA31	CA	CCA4.2	EixCc	EixCc	Cc - Ei	F1
CA32	CA	CCA24.1	EixCc	-	Cc - Ei	-
CA33	CA	CCA4.1	EixCc	-	Cc - Ei	-
CA36	CA	CCA4.1	EixCc	EixCc	Cc - Ei	F1
CE04	CE	CCA4.2	EixCc	EixCc	Cc - Ei	F1
CE32	CE	Hap F	EixLo	Ei/Lo	Lo - Ei	-
CE43	CE	Hap F	EixLo	Ei/Lo	Lo - Ei	-
CE51	CE	CCA4.2	EixCc	EixCc	Cc - Ei	F1
ES05	ES	Hap F	EixLo	-	Lo - Ei	-
UY01	UY	CCA4.2	EixCc	Ei	EixCc - Ei	F1xEi
UY05	UY	CCA4.2	EixCc	Ei	EixCc - Ei	F1xEi
*F: female – M: male.

## Discussion

In this work, we used mtDNA (D-Loop) and nDNA (RAG1, RAG2 and CMOS) markers to investigate hybridization in immature marine turtles in the South Western Atlantic, and identified 22 hybrid turtles in 270 samples (8.1%), with 60% of them occurring at the extreme south of their distribution (South Brazil and Uruguay). A previous study conducted along the Brazilian coast with immature turtles identified as *E. imbricata* found four hybrids with *C. caretta* through mtDNA analysis (Proietti *et al.*, 2014b).

With the increase in geographical coverage and sample number, we identified 17 hybrid Ei x Cc on the coast of the South Western Atlantic, based on mtDNA. The highest frequency of these hybrids occurred in Uruguay, which presented three hybrids in eight samples (37.5%), followed by Cassino, where 10 out of the 33 turtles analysed (30%) had *C. caretta* haplotypes. Proietti *et al.* (2014a) hypothesized that the high occurrence of hybrid turtles in temperate areas could be due to the adoption of the behavior of *C. caretta*, which occupies colder regions (Wallace *et al.*, 2010) than *E. imbricata*, which prefers tropical areas (León and Bjorndal, 2002; Mortimer and Donnelly, 2008). Our results corroborate this hypothesis, and we suggest that other methods such as telemetry or diet analysis should be used to confirm the differential behavior of hybrids.

Previous Ei x Cc records reported the occurrence of only one mtDNA haplotype, CCA4.2, the most common haplotype of females at Brazilian nesting grounds (Shamblin *et al.*, 2014). In the present work, two other haplotypes (CCA4.1 and CCA24.1) were identified for the first time in hybrids. These haplotypes are present only at nesting grounds in Brazil, but at lower frequency (Reis *et al.*, 2010). Based on haplotype frequencies, Shamblin *et al.* (2014) suggested a possible regionalization of *C. caretta* populations at the Brazilian coast, into two Regional Management Units (RUMs): 1) Sergipe and Bahia 2) and Espírito Santo and Rio de Janeiro. The hybrids we identified presented haplotypes most frequently found in the first RMU, and therefore we can consider that they most likely originate from the Bahia/Sergipe rookeries, reinforcing the observation that this is the main region where *C. caretta* and *E. imbricata* crossbreed.

We found three *E. imbricata* x *L. olivacea* hybrids, a type of cross that had not yet been reported for immature marine turtles along the South Western Atlantic coast. These hybrids presented the F haplotype, described by Bowen *et al.* (1998) as characteristic of *L. olivacea* and originating from nesting areas of the Atlantic Ocean (in descending order of frequency: Suriname, Brazil and Guinea Bissau). Rookeries for this species in Brazil are concentrated between Bahia and Alagoas, with higher density in Sergipe state (Castilhos *et al.*, 2011). Lara-Ruiz *et al.* (2006) observed two hybrids with haplotype F when analyzing the mtDNA of 119 adult *E. imbricata* samples from the north coast of Bahia. Vilaça *et al.* (2012) reported two occurrences of Ei x Lo at the coast of Bahia among 121 female *E. imbricata*, and concluded that all were the result of the crossing between a male *E. imbricata* and a female *L. olivacea* (F1). In addition, the authors state that these individuals showed no signs of introgression; therefore, they may be infertile or generated from rare hybridization events. The rarity of *E. imbricata* and *L. olivacea* hybrids can be explained by both species presenting their highest reproductive population densities in different areas: olive ridleys nest mostly in Sergipe state, reducing the probability of crossings between them. Sanches and Bellini (1999) observed that morphology may also influence this reproduction, since adult *L. olivacea* are smaller (mean CCL 73 cm; Silva *et al.*, 2007) than *E. imbricata* (mean CCL 97 cm; Marcovaldi *et al.*, 1999), which is in turn more similar to *C. caretta* (mean CCL 103 cm; Marcovaldi and Chaloupka, 2007).

The analysis of both mtDNA and nDNA increased the number of hybrid sea turtle detections at the South Western Atlantic coast. Based on mtDNA alone 20 hybrids were identified, and nDNA analysis revealed two additional hybrids not identified by morphology or mtDNA. In addition, with the analysis of three nDNA markers, it was possible to infer that 50% of these hybrids were first generation and 36% were backcrossed between hybrids and pure *E. imbricata* (>F1). The generations of two hybrids (CA23 and PF06) could not be directly determined, since they had morphology and mtDNA of the same species (*E. imbricata*) but nDNA with alleles of two different species (Ei x Cc). With these characteristics the individuals could be: F1, result of the unusual crossing between female *E. imbricata* and male *C. caretta*; or >F1, the result of a backcross between a hybrid male (Ei x Cc) and an *E. imbricata* female.

As mentioned above, most first generation hybrids (F1) result from mating between *C. caretta* females and *E. imbricata* males (Vilaça *et al.*, 2012). Crossings between F1 females and both pure parent species also occur, but crossings of pure females and hybrid males are apparently less frequent (Vilaça *et al.*, 2012, Soares *et al.*, 2018). Female *E. imbricata*, *C. caretta* and Ei x Cc hybrids have different nesting peaks, with pure *C. caretta* nesting earlier than hybrids, which nest earlier than pure *E. imbricata* (Soares *et al.*, 2017). Considering this, along with the gender bias of the reproductive groups, it is unlikely that the CA23 and PF06 individuals are the result of a cross between a female *E. imbricata* (pure) and a male *C. caretta* (F1). Since the *E. imbricata* nesting peak in Bahia occurs when most *C. caretta* males have already left the area, the likelihood of them reproducing is reduced. Indeed, this type of hybrid cross was observed in only one of the 82 females from Bahia analysed by Soares *et al.* (2017). This hybrid was also identified as *C. caretta* through morphological analysis, which did not occur in the individuals found in this study. Hybrid males (Ei x Cc) may show an intermediate reproductive period between pure parent species, as observed for hybrid females. In this case, they would encounter more *E. imbricata* females than the *C. caretta* males. Additionally, considering that backcrosses between Ei x Cc and pure *E. imbricata* have been observed more frequently than introgression with *C. caretta*, it is likely that CA23 and PF06 are a result of the cross between *E. imbricata* and hybrid males. Given our findings, we recommend that future studies sequence at least one nuclear loci in conjunction with the mtDNA to identify hybrids and potential introgression.

## Conclusions

Our study shows the importance of the combined use of mtDNA and nDNA markers in the evaluation of hybridization among marine turtles. Although mtDNA analysis is of paramount importance in the study of hybrids, nDNA analysis is also crucial for identifying generations/parental species, as well as for detecting hybrids in cases where mtDNA cannot (e.g. hybrid backcrosses with pure *E. imbricata*). This is confirmed by our observation of two hybrids that presented *E. imbricata* morphology and mtDNA, which could only be identified as Ei x Cc through nDNA. This observation may have been underestimated since we were not able to amplify the three nDNA markers for all samples. Another limitation was that the only nuclear marker amplified for all animals (RAG1) presents two shared haplotypes between *E. imbricata* and *L. olivacea*, and therefore it is not possible to infer conclusively about Ei x Lo hybrids with nDNA analysis. The hybridization events between *E. imbricata* and *L. olivacea* seems to be rare, but additional studies are necessary to identify species-specific haplotypes, allowing us to understand the generation and parental species of these hybrids.

Considering the high frequency of hybrids found in the South Western Atlantic, studies on the behavior, distribution, feeding strategy, migration and demography of hybrid turtles should be performed. Satellite tracking, stable isotope analysis and more comprehensive molecular tools such as genomics (e.g. SNP analysis), are techniques that would aid in understanding the process and better determining the ecological role of these hybrids, as well as their influence on marine turtle populations. According to Bohling (2016), the greatest concern regarding hybridization events is the extinction of genetic, phenotypic and/or evolutionary units due to a continuous hybridization process or hybrid vigor. Schwartz *et al.* (2007) suggest that this process be biologically and ecologically monitored in a continuous manner, providing a framework for determining and tracking the extent of hybridization, trends over time, and results of management strategies.

There are currently no guidelines for the management of hybrids and hybrid zones in Brazil, and most existing programs are focused on areas where the event has anthropogenic causes such as habitat change (Crispo *et al.*, 2011) and overexploitation (Bohling and Waits, 2015). To avoid the genetic extinction of pure species in the presence of a hybrid swarm, hybrid animals can be eliminated from the reproductive stock by eradication or sterilization. This type of action has already been carried out to control the hybridization beween the estuarine fish *Cyprinodon variegatus* and *Cyprinodon bovinus*, which was successful due to the limited geographic extension of the hybrid group (Echelle and Echelle, 1997). Considering the current endangered status of marine turtles and the high frequency of hybrids along the SWA coast, continuous monitoring should be carried out to assess the fitness, genetic integrity, and to detect the extent of changes in the gene pools of the involved populations. This is fundamental to evaluate if management of hybrid individuals should be considered, and ensure the conservation of SWA marine turtle populations.

## Supplementary material

The following online material is available for this article:

Table S1 - Individual haplotypes for the three nuclear loci and mtDNA.
