# Supplementary material for: Combined use of mitochondrial and nuclear genetic markers further reveal immature marine turtle hybrids along the South Western Atlantic
Source: Genet Mol Biol. 2020 Apr 27;43(2):e20190098. doi: 10.1590/1678-4685-GMB-2019-0098 (PMC7199923; doi:10.1590/1678-4685-GMB-2019-0098)
Supplement: Table S1 - [file 1415-4757-GMB-43-2-e20190098-s1.pdf]

Supplementary Material to "Combined use of mitochondrial and nuclear genetic markers further reveal immature marine turtle hybrids along the South Western Atlantic"

Table S1 – Individual haplotypes for the three nuclear loci and mtDNA. Colors represent probable species of origin from the haplotype: *E. imbricata* (green), *C. caretta* (red), or shared *E.imbricata*/*L.olivacea* (yellow). The row mtDNA denotes hybrid individuals classified from mtDNA data, while the row nuDNA denotes individuals classified as hybrids from nuclear data. Samples with indication of introgression/backcrossing are shown in bold. Region and site abbreviations follow Figures 1 and 3.

Sample	Site	Region	RAG1.1	RAG1.2	RAG2.1	RAG2.2	CMOS.1	CMOS.2	HAP mtDNA	mtDNA	nDNA
AB01	AB	AB	1	3					EiA01		
AB02	AB	AB	3	3	5	5			EiA32		
AB03	AB	AB	1	3	5	5			EiA01		
AB04	AB	AB	3	3	5	5			EiA01		
AB05	AB	AB	3	3	5	5			EiA01		
AB06	AB	AB	3	3	5	5	3	3	EiA01		
AB07	AB	AB	3	3	5	5			EiA01		
AB08	AB	AB			5	5			EiA01		
AB09	AB	AB	3	3	5	5			EiA01		
AB10	AB	AB	3	3	5	5			EiA01		
AB11	AB	AB	3	3	5	5			EiA01		
AB12	AB	AB	3	3	5	5			EiA01		
AB13	AB	AB	3	3	5	5			EiA01		
AB14	AB	AB							EiA01		
AB15	AB	AB	3	3	5	5			EiA01		
AB16	AB	AB	3	3	5	5			EiA01		
AB17	AB	AB	3	3	5	5	3	3	EiA01		
AB18	AB	AB	3	3	5	5			EiA01		
AB19	AB	AB	4	3					EiA01		
AB20	AB	AB	3	3					EiA01		
AB21	AB	AB	3	3	5	5			EiA01		
AB22	AB	AB	3	3	5	5			EiA01		
AB23	AB	AB							EiA01		
AB24	AB	AB							EiA01		
AB25	AB	AB			5	5	3	3	EiA01		
AB26	AB	AB							EiA01		
AB27	AB	AB	1	3					EiA01		
AB28	AB	AB							EiA01		
AB29	AB	AB	3	3	5	5			EiA01		
AB30	AB	AB							EiA01		
AB31	AB	AB							EiA01		

Sample	Site	Region	RAG1.1	RAG1.2	RAG2.1	RAG2.2	CMOS.1	CMOS.2	HAP mtDNA	mtDNA	nDNA
AB32	AB	AB							EiA62		
AB33	AB	AB							EiA01		
AB34	AB	AB							EiA01		
AB35	AB	AB							EiA01		
AB36	AB	AB							EiA01		
AB37	AB	AB							EiA62		
AB38	AB	AB							EiA24		
AB39	AB	AB							EiA01		
AB40	AB	AB							EiA01		
AB41	AB	AB							EiA62		
AB42	AB	AB							EiA01		
AB43	AB	AB	3	3					EiA01		
AB44	AB	AB	3	3					EiA09		
AB45	AB	AB							EiA01		
AB46	AB	AB							EiA01		
AB47	AB	AB							EiA01		
AB48	AB	AB							EiA01		
AB49	AB	AB							EiA01		
AB50	AB	AB							EiA01		
AB51	AB	AB							EiA01		
AB52	AB	AB							EiA01		
AB53	AB	AB	3	3					EiA01		
AB54	AB	AB							EiA01		
AB55	AB	AB							EiA01		
AB56	AB	AB							EiA01		
AB57	AB	AB							EiA01		
AB58	AB	AB							EiA01		
AB59	AB	AB							EiA01		
AB60	AB	AB							EiA01		
AB61	AB	AB							EiA01		
AB62	AB	AB							EiA01		
AB63	AB	AB							EiA01		
AB64	AB	AB							EiA01		
AB65	AB	AB							EiA32		
AB66	AB	AB							EiA01		
AB67	AB	AB	3	3					EiA01		
AB68	AB	AB	3	3					EiA61		
AB69	AB	AB	1	3					EiA01		
AB70	AB	AB	3	3					EiA62		
AB71	AB	AB	3	3					EiA01		
AB72	AB	AB	3	3	5	5			EiA01		
AB73	AB	AB	3	3	5	5	3	3	EiA01		
AB74	AB	AB	3	3	5	5	3	3	EiA01		
AB75	AB	AB	3	3	5	5			EiA01		

Sample	Site	Region	RAG1.1	RAG1.2	RAG2.1	RAG2.2	CMOS.1	CMOS.2	HAP mtDNA	mtDNA	nDNA
AL01	AL	AL							EiA01		
AL02	AL	AL							CC-A4.2	CC	
AL03	AL	AL	3	3					EiA01		
AL04	AL	AL							EiA01		
AL05	AL	AL	3	3					EiA01		
AL06	AL	AL	3	3			3	10	EiA01		
AP01	AP	BA	3	3	5	5			EiA01		
AP02	AP	BA							EiA01		
AP03	AP	BA	3	3	5	5			EiA01		
AP04	AP	BA	3	3	5	5			EiA76		
AP05	AP	BA	3	3					EiA01		
AP06	AP	BA	3	3	5	5			EiA01		
AP07	AP	BA							EiA01		
AP11	AP	BA							EiA01		
AP28	AP	BA							EiA01		
AP42	AP	BA	3	3					EiA01		
AP43	AP	BA			5	5	3	3	EiA01		
AP44	AP	BA			5	5	3	10	EiA01		
AP49	AP	BA	4	3	5	5			EiA09		
AP53	AP	BA							EiA01		
AP54	AP	BA	3	3	5	5			EiA09		
AP55	AP	BA	3	3	5	5	3	3	nan		
AP56	AP	BA	2	3					CC-A4.2	CC	EixCc
AP57	AP	BA							EiA01		
AP58	AP	BA			5	5			EiA01		
AP59	AP	BA							EiA01		
AP60	AP	BA							EiA01		
AP61	AP	BA							EiA01		
AP62	AP	BA							EiA62		
AP63	AP	BA							EiA32		
AP64	AP	BA							EiA32		
ARV1	ARV	SC	3	3					EiA01		
ARV2	ARV	SC	3	3			3	5	EiA01		
ARV3	ARV	SC	3	3					EiA01		
ARV4	ARV	SC	3	3	5	5			EiA62		
ARV5	ARV	SC	3	3	5	5	3	3	EiA62		
ARV6	ARV	SC	3	3	5	5			EiA62		
ASP01	ASPSP	ASP							EiA01		
ASP02	ASPSP	ASP							EiA01		
ASP03	ASPSP	ASP							EiA01		
ASP04	ASPSP	ASP							EiA01		
ASP05	ASPSP	ASP							EiA01		

Sample	Site	Region	RAG1.1	RAG1.2	RAG2.1	RAG2.2	CMOS.1	CMOS.2	HAP mtDNA	mtDNA	nDNA
ASP06	ASPSP	ASP							EiA62		
ASP07	ASPSP	ASP							EiA62		
ASP08	ASPSP	ASP							EiA01		
ASP09	ASPSP	ASP							EiA62		
ASP10	ASPSP	ASP							EiA01		
ASP11	ASPSP	ASP							EiA92		
ASP12	ASPSP	ASP							EiA23		
CA01	CA	CA	3	3	5	5	3	10	EiA01		
CA02	CA	CA							EiA01		
CA03	CA	CA							EiA01		
CA04	CA	CA							CC-A4.2	CC	
CA06	CA	CA							EiA01		
CA08	CA	CA	3	3					EiA01		
CA09	CA	CA							EiA01		
CA10	CA	CA	3	3	5	5	5	5	EiA01		
CA11	CA	CA	3	3	5	5	3	3	EiA01		
**CA12**	CA	CA	3	3					CC-A4.2	**CC**	
CA13	CA	CA	3	3					EiA62		
**CA14**	CA	CA	3	3	5	5	3	3	CC-A4.2	**CC**	
CA15	CA	CA	3	3	5	5	3	3	EiA01		
CA16	CA	CA	3	3	5	5	3	3	EiA01		
CA17	CA	CA	3	3	5	5	3	3	EiA62		
CA18	CA	CA	3	3	5	5	3	3	EiA01		
CA19	CA	CA	3	3	5	5	3	10	EiA01		
CA20	CA	CA	3	3	5	5	3	10	EiA01		
CA21	CA	CA			5	5			nan		
**CA22**	CA	CA	3	3					CC-A4.2	**CC**	
CA23	CA	CA	2	3	2	5	2	3	EiA01		EixCc
CA24	CA	CA	2	3	2	5	1	10	CC-A4.2	CC	EixCc
CA25	CA	CA	2	3					CC-A4.2	CC	EixCc
CA26	CA	CA	3	10					EiA01		
CA27	CA	CA							EiA01		
CA28	CA	CA							EiA01		
CA29	CA	CA							EiA01		
CA30	CA	CA							EiA01		
CA31	CA	CA	2	3					CC-A4.2	CC	EixCc
CA32	CA	CA							CC-A24.1	CC	
CA33	CA	CA							CC-A4.1	CC	
CA34	CA	CA							EiA01		
CA35	CA	CA							EiA01		
CA36	CA	CA	2	3	2	5			CC-A4.1	CC	EixCc
CE01	CE	CE							EiA01		

Sample	Site	Region	RAG1.1	RAG1.2	RAG2.1	RAG2.2	CMOS.1	CMOS.2	HAP mtDNA	mtDNA	nDNA
CE02	CE	CE							EiA01		
CE03	CE	CE							EiA11		
CE04	CE	CE	2	3					CC-A4.2	CC	EixCc
CE06	CE	CE							EiA01		
CE07	CE	CE							EiA01		
CE08	CE	CE							EiA01		
CE09	CE	CE							EiA01		
CE10	CE	CE							EiA01		
CE11	CE	CE							EiA01		
CE12	CE	CE							EiA01		
CE13	CE	CE							EiA62		
CE14	CE	CE							EiA01		
CE15	CE	CE							EiA01		
CE16	CE	CE							EiA01		
CE17	CE	CE							EiA01		
CE18	CE	CE	3	3					EiA01		
CE19	CE	CE							EiA01		
CE20	CE	CE							EiA01		
CE21	CE	CE	3	3			3	5	EiA01		
CE22	CE	CE							EiA01		
CE23	CE	CE	1	3					EiA01		
CE24	CE	CE	3	3			3	3	EiA01		
CE25	CE	CE							EiA01		
CE26	CE	CE	3	3					EiA01		
CE27	CE	CE	3	3					EiA01		
CE28	CE	CE	3	3	5	5	3	3	EiA62		
CE29	CE	CE							EiA01		
CE30	CE	CE	3	3					EiA01		
CE31	CE	CE	3	3			3	5	EiA62		
CE32	CE	CE	1	1					hap F short	LO	
CE33	CE	CE	3	3					EiA01		
CE34	CE	CE	3	3					EiA32		
CE35	CE	CE	3	3					EiA01		
CE36	CE	CE							EiA01		
CE37	CE	CE	3	3					EiA01		
CE38	CE	CE	3	3					EiA32		
CE39	CE	CE							EiA01		
CE40	CE	CE	3	3					EiA01		
CE41	CE	CE	3	3					EiA01		
CE42	CE	CE							EiA01		
CE43	CE	CE	1	3					hap F short	LO	
CE44	CE	CE	3	3	5	5			EiA01		
CE45	CE	CE	3	3					EiA01		
CE46	CE	CE	3	3					EiA01		

Sample	Site	Region	RAG1.1	RAG1.2	RAG2.1	RAG2.2	CMOS.1	CMOS.2	HAP mtDNA	mtDNA	nDNA
CE47	CE	CE	3	3	5	5			EiA01		
CE48	CE	CE							EiA01		
CE49	CE	CE	3	3	7	7	3	3	EiA01		
CE50	CE	CE			5	5			EiA01		
CE51	CE	CE	2	3	2	5			CC-A4.2	CC	EixCc
CE52	CE	CE							EiA01		
CE53	CE	CE							EiA01		
CE54	CE	CE							EiA01		
CE55	CE	CE							EiA01		
CE56	CE	CE							EiA01		
CE57	CE	CE							EiA01		
ES01	ES	ES							EiA01		
ES02	ES	ES							EiA01		
ES03	ES	ES							EiA01		
ES04	ES	ES							EiA01		
ES05	ES	ES							hap F short	LO	
ES06	ES	ES	3	3					nan		
ES07	ES	ES							EiA01		
ES09	ES	ES							EiA01		
ES10	ES	ES	3	3	5	5			EiA32		
ES11	ES	ES	3	3	5	5	3	3	EiA01		
ES12	ES	ES	3	3	5	5			EiA01		
PF01	PF	BA							EiA01		
PF02	PF	BA							EiA01		
PF03	PF	BA			5	5			EiA62		
PF04	PF	BA	3	3	5	5			EiA01		
PF05	PF	BA	3	3	5	5			EiA01		
PF06	PF	BA	2	3	2	5			EiA01		EixCc
PF07	PF	BA	3	3	5	5	3	5	EiA01		
PF08	PF	BA	4	3	5	5			EiA01		
PF09	PF	BA	3	3	5	5			EiA32		
PF10	PF	BA	3	3	5	5			EiA01		
PF11	PF	BA	3	3	5	5			EiA32		
PF12	PF	BA	3	3	5	5	3	3	EiA28		
PF13	PF	BA			5	5			EiA01		
PF14	PF	BA	3	3	5	5			EiA32		
PF15	PF	BA	3	3	5	5			EiA01		
PF16	PF	BA			5	5	3	5	EiA01		
PF17	PF	BA	3	3	5	5	3	10	EiA01		
PF18	PF	BA	3	3	5	5	9	9	EiA01		
PF19	PF	BA							EiA01		
PF20	PF	BA	3	3	5	5	3	3	EiA01		
PF21	PF	BA	1	3	5	5			EiA32		
PF22	PF	BA	3	3	5	5			EiA01		

Sample	Site	Region	RAG1.1	RAG1.2	RAG2.1	RAG2.2	CMOS.1	CMOS.2	HAP mtDNA	mtDNA	nDNA
PF23	PF	BA	3	3					EiA01		
PF24	PF	BA							EiA01		
SA01	SA	BA							EiA01		
SA02	SA	BA							EiA01		
SA03	SA	BA			5	5			EiA01		
SA04	SA	BA	3	3					EiA01		
SA05	SA	BA	3	3	5	5			EiA01		
SA06	SA	BA			5	5			EiA01		
SA07	SA	BA							EiA01		
SA08	SA	BA							EiA01		
SA09	SA	BA							EiA01		
SA10	SA	BA							CC-A4.2	CC	
SA11	SA	BA							EiA01		
SA12	SA	BA							EiA01		
SA13	SA	BA							EiA01		
**UY01**	UY	UY			5	5			Cc-A4.2	**CC**	
UY02	UY	UY			5	5			EiA32		
UY03	UY	UY	1	3	5	5			EiA01		
**UY05**	UY	UY			5	5			Cc-A4.2	**CC**	
UY06	UY	UY	3	3	5	5			EiA01		
UY07	UY	UY							Cc-A4.2		
UY08	UY	UY	3	3	5	5			EiA01		
UY09	UY	UY	3	3	5	5			EiA01		
UY10	UY	UY	3	3					nan		
